# Self-Care Associated with Home Exercises in Patients with Type 2 Diabetes Mellitus

**DOI:** 10.1371/journal.pone.0114151

**Published:** 2014-12-05

**Authors:** Denise H. Iunes, Carmélia B. J. Rocha, Nathália C. S. Borges, Caroline O. Marcon, Valéria M. Pereira, Leonardo C. Carvalho

**Affiliations:** 1 Federal University of Alfenas, Alfenas, Brazil; 2 Health of the Municipal State of Alfenas, Alfenas, Brazil; Weill Cornell Medical College Qatar, Qatar

## Abstract

The objective of this study was to verify self-care guidelines together with lower limb home exercises alter ankle and foot plantar pressure and alignment in patient with Type 2 Diabetes Mellitus (DM) measuring health and sociodemographic factors. The health factors analyzed were sensitivity and circulation aspects, risk rating, and neuropathy symptom score, ankle and foot alignment (photogrammetry), plantar pressures, and postural stability (baropodometry) before and after administering these guidelines and home exercises in 97 patients type 2 DM during 10 months. The self-care guidelines and exercises changed the forefoot alignment (Right Foot – Initial vs Final, *p* = 0.04; Left Foot, *P*<0.01), the center of the force displacement in the mediolateral (Right Foot - Initial versus Final, *p* = 0.02; Left Foot, *P*<0.01), and the anterior-posterior (Right foot - Initial versus Final, *p* = 0.01) direction, and body balance (Initial versus Final, *p* = 0.02). There was no change in the remaining assessed parameters. Self-care associated with the guidelines for home exercises for the lower limbs in patients with type 2 DM are effective in maintaining and improving the alignment of the feet, mediolateral stability and prevention of complications.

**Trial Registration:**

The Brazilian Clinical Trials Registry RBR-8854CD

## Introduction

Foot alterations related to diabetic neuropathy include atrophy of the foot muscles with an imbalance between the flexor and extensor muscles, which produces changes in claw toes and weight-bearing [1], [2] and the consequent calluses. These alterations also predispose patients to ulceration [3], loss of muscle strength [1], decreased ankle mobility [4] and changes in gait [3], [5]. Another complication of diabetic neuropathy is postural instability, which predisposes patients to fall [6]. This postural instability is attributed to a decrease in the peripheral sensory system that occurs with diabetic neuropathy [6].

The amount of amputations due to diabetic foot ulcers remain significant in both developed and developing countries [7]. However, when discussing self-care, there is a tendency to valorize glycemic control by health professionals [8]. It is well established that the problems arising from diabetic foot ulcerations are preventable if specialized care is provided [9].

The muscle impairment and the posture changes of the ankles and feet are well described in the literature [4], [10], [11], but there is a lack of study into whether the use unsupervised home exercises for the feet and ankles may benefit diabetes patients. Thus, we analyzed the diabetes patients' adherence to the guidelines for self-care and home exercises, and additionally, we evaluated the alignment of the feet by photogrammetry, the body balance and plantar pressures by baropodometry and other clinical aspects.

## Methods

The protocol for this trial and supporting CONSORT checklist are available as supporting information; see Checklist S1 and Protocol S1.

### Sample

This study is characterized as prospective and quasi-experimental (The Brazilian Clinical Trials Registry RBR-8854CD). All individuals with Type 2 DM were recruited at a Basic Health Unit of the City of Alfenas where they had medical monitoring. The inclusion and exclusion criteria were evaluated by clinical examination.

The inclusion criteria were: the clinical diagnosis of patient with Type 2 DM for more than 5 years, the use of oral hypoglycemic agents and/or insulin and the performance of routine medical monitoring.

The exclusion criteria were: Individuals with Type 2 DM with very high risk i.e., those with ulcers and/or previous amputations, the clinical diagnosis of hemiplegy, paraplegy, or Parkinson's disease; limb amputation; a history of alcohol or drug abuse; a herniated disc; leprosy; severe arthritis that prevents brisk walking; dementia; intellectual disability; and other psychiatric disorders. The first criteria were set because it would take the change in weight distribution on the lower limbs and the three last criteria because it could interfere with the understanding of guidelines for exercise and self-care.

The participant recruitment and follow-up occurred between the period of May 2011 to December 2012.

**Figure 1:**
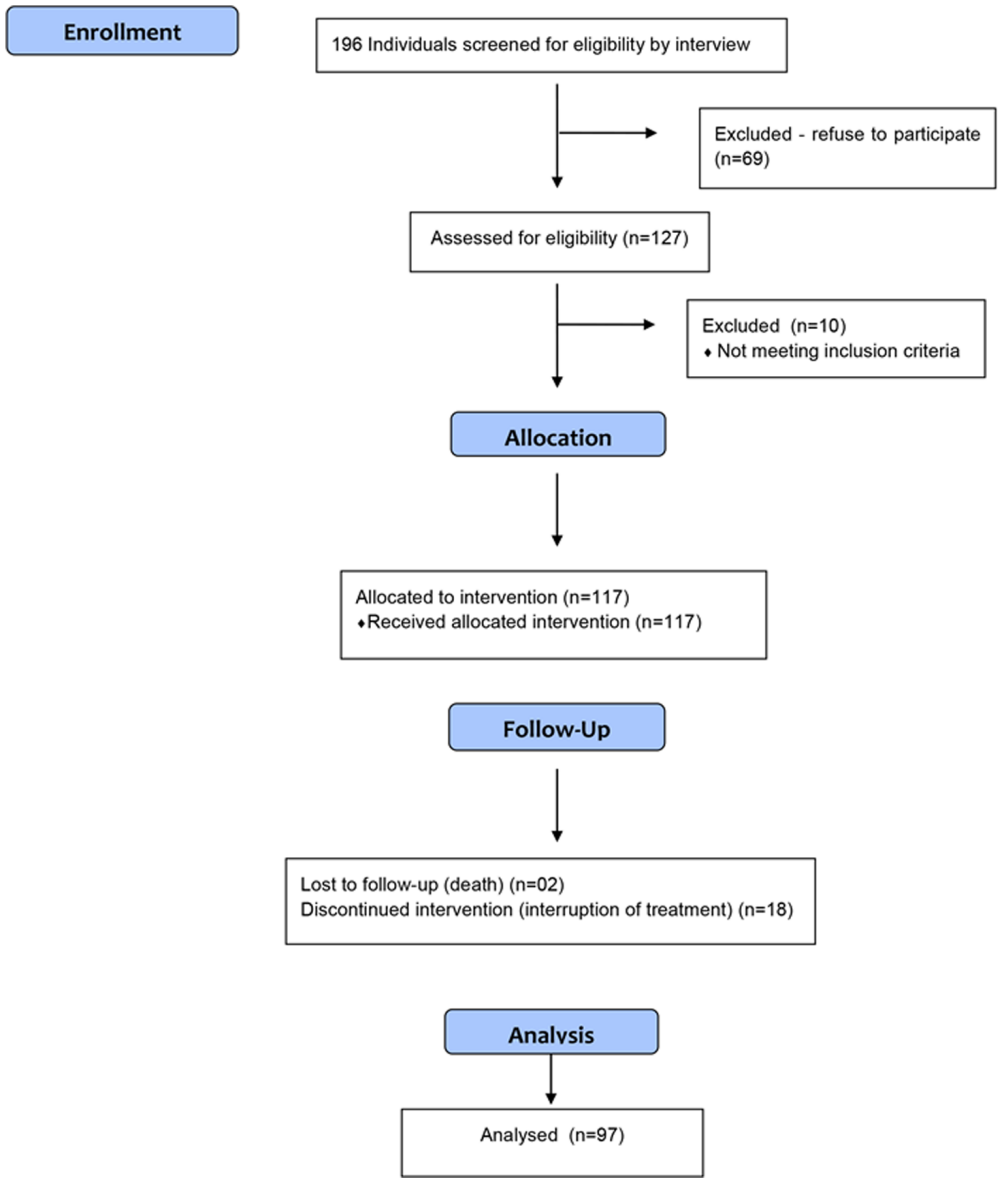
Consort flowchart.

One hundred and ninety-six patients were selected and distributed as shown in Figure 1.

All individuals (n = 97, mean age 62.12±11.31 years, 56.0% women, duration of diabetes 13.89±7.57 years, BMI 17.64±3.80 Kg/m^2^, HbA_1c_ 7.9±1.0% or 63±−12 mmol/mol) initially received individual verbal guidance as well as an explanatory leaflet about self-care and lower limb exercises (Table 1) and had follow-up examinations for 10 consecutive months; they were instructed to return at least once per month. All evaluation and interventions were performed by a single investigator.Only 22.7% of the individuals had previously received some counseling or assessment on self-care for their feet by member(s) of the nursing staff before the beginning of the study. We observed that the average number of follow-up visits attended during the 10 month follow-up period was 5.12±3.14 times. However, 53.6% of the sample attended more than five follow-up sessions.

**Table 1:** Check list for the exercise and self-care guidance used in the follow-up to the individuals.

Detail	Exercises		Self-care	
**01**	Dorsiflexion and plantar flexion, lying (pumping exercise). Verbal Command: “Lying, raise and lower your foot tip 30 times”.	()	Use of closed shoes [12], [13].	()
**02**	Feet circumduction, lying (pumping exercise). Verbal Command: “Make legs rotate 30 times to the right, then 30 times to the left”.	()	Do not walk barefoot [12], [14].	()
**03**	Dorsiflexion and plantar flexion, sitting (pumping exercise). Verbal Command: “While seated, lift your heels off the ground and lower and lift the tips of the feet 30 times”.	()	Cut the nails properly [14].	()
**04**	Stretching flexors of the toes. Verbal Command: “Rest your feet flat and supported, stretch your toes to the maximum and count to 50”.	()	Use mirror for self-examination [8], [13].	()
**05**	An exercise to strengthen the intrinsic muscles of the feet using a towel - Verbal Command: “Extend a large towel on the ground, rest both feet on it, and then pull the toes with the towel”.	()	Proper drying of the feet [12].	()
**06**	Proprioceptive exercise of the foot plant with a curly ball. Verbal Command: “For two minutes for each foot, slide your feet on a curly short small ball”.	()	Wearing cotton socks [7].	()
**07**	Exercise to strengthen the intrinsic muscles of the feet with the use of marble. Verbal Command: “Hold 30 marbles with the toes of each foot”.	()	Use moisturizer on legs [12], [14].	()
**08**	Free active exercise for hip abductors in standing position. Verbal Command: “While standing, close your legs for 30 repetitions”.	()	Non-application of moisturizer between toes [12], [14].	()
**09**	Free active exercise for hip abductors in standing position. Verbal Command: “While standing, open the legs for 30 repetitions”.	()	Do not use abrasives or sandpaper [12].	()
**10**	Stretching triceps surae. Verbal Command: “Standing with one foot forward and one back and the heels in contact with the ground, count to 50”.	()		
**11**	Strengthening triceps surae. Verbal Command: “Stand on tiptoe up and down slowly for 30 repetitions”.	()		

A checklist was used (Table 1) to assess the patients' adherence to the guidelines. The researcher marked positive (+) when the volunteer claimed to have performed a particular orientation and negative (−) when the volunteer had not. This project was approved by the Research Ethics Committee of the Federal University of Alfenas.

All participants provided informed, written consent before participation and were informed about the study's objectives.

### Measures

The data were collected by the same examiner before and after the follow-up period and the practical guidelines on the management and prevention of diabetic foot ulcerations were followed [12]. Sociodemographic and health factors were collected, including age, gender, BMI, duration of diabetes, medication used for glycemic control (insulin, oral hypoglycemic or insulin and oral hypoglycemic), and factors associated with diabetes, such as smoking, alcohol consumption, hypertension, skin aspects, toe deformities, muscle strength of the toes' flexors and extensors, hallux extensor and eversion [15], sensitivity (Semmes-Weinstein 10 g) [12], joint mobility and circulation aspects (the presence of edema as well as tibial and dorsal pedis pulses).

Risk ratings were determined from the data obtained in the initial evaluation [16] and were based on the following considerations: low risk, individuals without sensory neuropathy; moderate risk, individuals with sensory neuropathy; and high risk, those with sensory neuropathy and signs of peripheral vascular disease and/or foot deformity. Individuals with very high risk were excluded from the study, i.e., those with ulcers and/or previous amputations.

The neuropathy symptoms score (NSS) created by Young et al. [17] translated to and validated for Portuguese [11] was applied. This scale allowed the assessment of the pain or discomfort symptoms in the legs, their locations, the time of day that they are aggravated and the maneuvers that relieved the painful symptoms.

**Figure 2:**
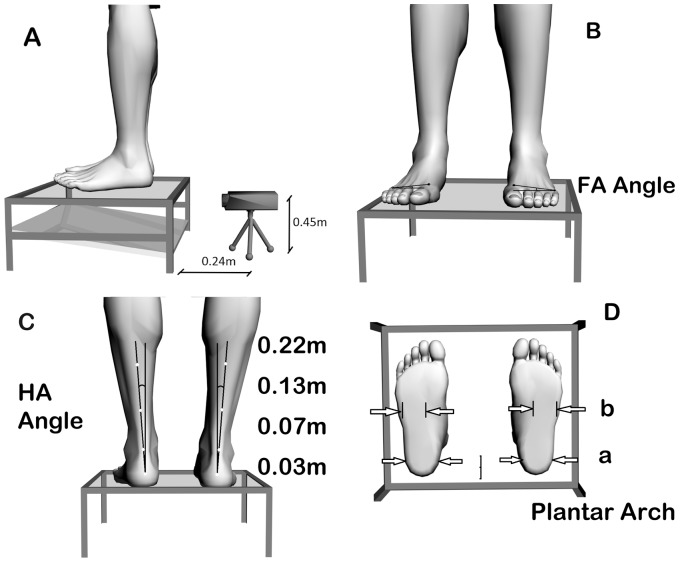
Photogrammetric analysis (A) of the forefoot angle (B), the rear foot angle (C) and the plantar arch (D).

We also collected photographs of the feet and ankles' anterior (Figure 2A and 2B), posterior (Figure 2C) and plant for photogrammetric analysis (Figure 2D). The individuals were initially positioned in a standing position with bare feet on a podoscope. Then, the photographic records were made with a digital camera, *Nikon Coolpix* 10 megapixels, positioned on a tripod that had been previously leveled in a perpendicular position (Object - camera distance  = 0.24 m; Camera height  = 0.45 m) in front of the photographed individual. The following anatomical landmarks were marked on the body of the individuals using self-adhesive labels: the heads of the first and fifth metatarsals; the tuberosity of the calcaneus at 0.03 m above the ground, and other markers, respectively positioned at 0.07, 0.13 and 0.22 m above the ground. These markings were used to check the alignment of the Achilles tendon [18].

CorporisPro (Datahominis Technology, Brazil) was used to analyze the images to verify the angular measurements, and Postural Assessment Software (SAPo) was used to analyze the linear measurements of the plantar arch (Figure 2D) [19].

For each analyzed angle, three consecutive measurements were performed, and the arithmetic mean was calculated. The photographs were analyzed by a researcher blinded to whether the images were from the beginning or end of the study.

The following pattern was adopted in the angular analysis of the photographs:

Forefoot - Pronation <0° (Normal) <Supination [20]; Rearfoot – Calcaneal Varus <0° to 5° (Normal) <Calcaneal Valgus [21]; Plantar arch – Cavus Foot <0.003 to 12.01 m (Normal) < Flat foot [19].

In addition to this, static as well as dynamic baropodometric records were taken with the aid of a FootWork Pressure Plate (FPP) (IST Informatique, France) [22]. The FPP (Size: 0.45 m×0.58 m) used in this study contained 2704 Sensors type capacitive in order to measure the displacement of center of pressure (COP) and the foot displacements medial-lateral (ML) and anterior-posterior (AP) as well as plantar pressures mean and maximum, contact area and contact time.

An ethyl vinyl acetate (EVA) marker, 0.08 m in width by 0.20 m in length, positioned on the platform was used to standardize the position of the feet. Once the volunteer was positioned, the marker was removed and the volunteer was asked to keep his eyes open and stare at a white wall. The collection time was 20 seconds.

To perform the dynamic analysis, the volunteer was instructed to walk for a five meter distance on an EVA catwalk, with the baropodometer positioned in the center of the catwalk.

### Statistical Analysis

The statistical analyses were performed using SPSS 20.0 for Windows. The values were expressed as the means ± SD or percentages. The data obtained were subjected to the Kolmogorov-Smirnov test to assess the normality of the sample. After this analysis, the comparisons between the initial and the final tests were conducted, where the sample's paired t test was normal and the Wilcoxon two-sample test did not have normality.

To analyze nominal data, *X* ^2^ tests were used for parametric data and McNemar tests for nonparametric data. The F-test was used for analysis of variance ratio.

Taking forefoot as the main variable, a power effect of 0.95 and an effect size of 0.72 (α = 0.05). The required sample was estimated in 27 subjects. The *GPower* 3.1.7 software (*Franz Faut*, *Universität Kiel Germany*, 2008) was used for analyses.

For all the tests, *P* was <0.05, which was considered to be statistically significant.

## Results

The sociodemographic and clinical characteristics of both samples are displayed in Table 2. It was observed that, despite the time with diabetes, few individuals showed significant physical changes in anhydrase, temperature or edema. In contrast, the majority of patients showed a decrease in or absence of hair growth as well as the presence of varicose veins and mycoses.

**Table 2:** Patient characteristics.

ITEMS MEASURED		n = 97	
Body Mass (kg)			72.34±6.01
Height (m)			1.55±0.60
Diabetes Time (years)			13.89±7.57
Sex	Woman		60.8
	Man		39.2
Glycemic Control	Insulin		6.2
	Oral hypoglycemic		79.4
	Insulin and hypoglycemic		14.4
Smoker			9.3
Alcohol Abuse			8.3
Hypertension			73.2
Hairiness	Normal		20.6
	Decreased		50.5
	Absent		28.9
Anhidrosis			36.1
Skin color	Normal		93.8
	Cyanosis		5.2
	Erythema		1.0
Skin Temperature	Normal		97.9
	Decreased		2.1
Presence of mycosis			46.4
Edema			19.6
Varices	In any limb		18.6
	Only in the leg		29.9
	Varicosities		5.2
	Formation of us		1.0
Foot deformities	Absent		23.7
	Present		76.3

Data are means ± SD or % indicated.

The frequencies at which the individuals confirmed performing the guidelines of the conduct exercises and self-care are shown in Table 3. On average, the individuals attended 5.12±3.14 follow-up appointments during the monitoring period.

**Table 3:** Frequency of adherence to the guidelines for the exercises and self-care of the feet (n: 97).

Detail	Exercises	*F*	*p* value	Self-care	*F*	*p* value
01	4.73±3.17	1.02	0.90	3.72±3.24	1.16	0.47
02	4.46±3.06	1.05	0.81	3.95±3.07	1.07	0.73
03	4.29±3.09	1.03	0.89	3.99±3.04	1.02	0.91
04	3.99±3.23	1.06	0.77	2.94±3.04	1.12	0.55
05	4.16±3.11	1.01	0.94	4.31±3.08	1.01	0.94
06	3.67±3.26	1.08	0.69	2.73±3.01	1.04	0.85
07	3.41±3.06	1.05	0.81	3.69±3.03	1.02	0.92
08	2.71±2.93	1.14	0.49	3.85±3.09	1.12	0.57
09	2.94±3.03	1.07	0.74	3.62±3.07	1.11	0.60
10	2.82±2.87	1.19	0.38			
11	2.18±2.87	1.19	0.39			

Data are means ± SD indicated. The numbers in detail are the same as table 1. *versus* mean attendance - variance ratio (*F*) ratio significance *P*<0.05. Variance ratio test (*F- test)*.

The results of the flexor toes, extensor toes, extensor hallucis and eversion strength tests did not change after the follow-up period (Initial *versus* Final; *P*>0.05).

Tactile sensitivity was assessed; at the beginning of the study, 27.0% of the sample showed sensitivity changes at some of the assessed points, and 22.0% maintained this change at the end of the study (*Χ* ^2^ test, *p* = 0.19).

For the peripheral pulse, it was observed that at the beginning of the study, 72.0% and 69.0% (right and left tibialis pulses, respectively) of the patients, and at the end of the study 71.0% and 73.0% (*Χ* ^2^ test, *p* = 0.85) of the patients, had reduced or absent peripheral pulses. As for the pedal pulse, in the beginning of the study it was observed that 53.0% and 68.0% (right and left) of the individuals showed the same change described above, and at the end of the study 56.0% and 68.0% of the individuals showed this change (*Χ* ^2^ test, *p* = 0.93).

At the beginning of the study, the risk rating analysis revealed that 50.0% of the sample had a low risk, 38.0% of the sample had a moderate risk and 12.0% of the sample had a high risk, whereas at the end of the study 66.0% of the sample had a low risk, 24.0% of the sample had a moderate risk and 10.0% of the sample had a high risk (*Χ* ^2^ test, *p* = 0.06).

A large portion of the individuals, 71.1%, had neuropathy symptoms (NSS) [11]; 20.6% of these were classified as mild neuropathic symptoms, 21.6% as moderate and 28.9% as severe.

The foot alignment analysis, performed by photogrammetry, is shown in Table 4. A reduction in the angle values of the forefoot of both members with improvement in supination was observed.

**Table 4:** Comparison of the alignment of the feet before (initial) and after (final) 10 monitoring sessions.

Measure	Right foot		*p* value	Left foot		*p* value
	Initial	Final		Initial	Final	
Forefoot (°)	7.15±2.69	6.61±2.46	0.04*	11.78±5.29	8.45±3.02	<0.01*
Rearfoot (°)	6.08±5.10	6.14±4.81	0.97	6.20±5.31	6.49±5.07	0.50
Plantar Arch (m)	0.39±0.26	0.46±0.24	0.35	0.39±0.28	0.45±0.24	0.24

Data are means ± SD indicated.

*Wilcoxon test with *P*<0.05.

Forty-five individuals underwent baropodometric evaluation. Table 5 presents the results of the static analysis before (initial) and after (final) 10 months of follow-up. There was an increase in the mean plantar pressure of the left foot associated with a reduction in the anteroposterior oscillation amplitude of the right foot. In both feet as well as the body, a decrease in the COP and mediolateral displacement amplitude was observed.

**Table 5:** Static (S) and dynamic (D) podometric analysis before and after 10 month follow-up (n = 45).

Parameters		Right foot		*p*	Left foot		*p*
		Initial	Final	value	Initial	Final	value
Mean pressure	S	30.40±8.82	31.38±6.86	0.08	29.42±7.84	31.38±6.86	0.02*
(Kpa)	D	108.85±26.47	106.89±25.50	0.67	109.83±23.54	111.79±25.50	0.52
Maximum pressure	S	103.95±43.15	101.01±33.34	0.54	94.14±34.32	100.03±25.50	0.21
(Kpa)	D	237.32±40.21	251.05±48.05	0.11	241.24±50.01	222.61±50.99	0.23
Contact Area	S	99.18±18.23	99.91±18.66	0.58	94.87±17.67	94.85±17.68	0.99
(cm^2^)	D	105.57±23.17	108.74±18.77	0.26	102.38±17.66	105.07±16.21	0.07
Contact time (ms)	D	1135.78±587.88	1109.33±364.46	0.70	1093.11±510.88	1126.44±391.07	0.20
ML(cm)	S	0.72±0.63	0.50±0.34	0.02*	0.86±0.66	0.49±0.29	<0.01*
AP (cm)	S	3.19±1.70	2.58±1.15	0.01*	3.01±1.34	2.80±1.30	0.42
		ML(cm)			AP(cm)		
Body (cm)	S	2.11±1.30	1.64±0.92	0.02*	2.73±1.38	2.38±1.05	0.10

Data are means ± SD indicated.

*Paired *t* test *P*<0.05. Center of pressure displacement in mediolateral (ML), anterior-posterior (AP) and body (DB) direction.

## Discussion

The results of this study indicate that the guidelines associated with self-care exercises change the alignment of the feet and reduce the amplitude of the lateral oscillation of the lower limbs and body.

It was found that foot evaluation as well as the administration of self-care guidelines are not frequent practices of health care workers, even though the importance of these practices for the prevention of diabetic foot complications has been established [7], [13], [23], [24]. In our study, only 22 individuals reported that their feet had been examined or that they had received some type of counseling by the nursing staff.

We observed that there was adherence of the patients to the provided guidelines, even though there was a restricted frequency of monthly attendance to follow-up appointments. We emphasize that an individualized application of the guidelines was adopted, and, despite a better understanding of the guidelines by the patients when recommendations are provided individually, counseling, even if in a group, is always important [23].

Among the provided self-care practice instructions, we observed lower adherence to the use of a mirror for self-examination, with a reported preference for the help of family members due to vision and mobility difficulties [25].

As to the use of closed shoes and cotton socks, many individuals stated they did not adopt these measures due to the sensation of heat and burning in the feet. We believe that because diabetes patients have higher plantar temperature variations [26], the perceived discomfort was greater, causing them not to follow this guideline.

None of the individuals had any complications in their feet during the monitoring period. Similar findings were observed when the patients received the same type of self-care guidance and were encouraged to practice regular exercise [7], [23], [24].

Diabetes results in severe weakness in the legs and alters the function of the feet [27], which explains the importance of exercising the lower limbs. However, no other studies were found that were geared toward specific exercises for the feet as was recommended in this study - only indications to perform regular exercises were reported [24]. Nevertheless, regular exercise, supervised by a professional, is of utmost importance, as long as improvements in muscle strength, mobility, peripheral pulses and risk ratings are not observed. An increase in muscle strength was not expected to occur because there was no use of load while carrying out the exercises.

Ankle and foot biomechanics are altered in diabetes patients regardless of the presence of neuropathy because there is a reduction in mobility [11], increase in plantar pressure [4] and change in hindfoot kinematics [10]. A large number of patients initially showed supination in the forefoot and valgus calcaneal tendon. These changes may predispose patients to an increased pressure on the fifth toe and the medial region of the heel, which are related to the risk of plantar ulceration [22]. There was no change in the mobility of the ankle or feet with the exercises used in this study. Though there was a better distribution of the left foot's mean plantar pressure and a decrease of supination in the forefoot of both feet. Related to this observation, it was also found that the plantar arches tended to normality. Such facts explain why both the static and dynamic evaluations by baropodometry did not change in most of the assessed parameters. We believe that this factor could have contributed to the prevention of complications, such as foot ulcers, especially in the forefoot, as long as the guidelines were maintained.

In this study, there was no comparison of the postural stability between patient with diabetes and patient without diabetes, as has been performed in other studies [6], which indicated a higher COP in patient with diabetes. However, it was observed that after 10 months of exercises there was an improvement in the peripheral sensitivity of the feet in both the lateral lower limbs, the body's COP and the anteroposterior right lower limb's COP (Table 5).

Peripheral arterial vascular impairment is very common but is still underestimated in diabetes patients; it should be a concern because it predisposes patients to co-morbidities, such as severe ischemia and amputations [28]. When the goal is to improve the peripheral pulses, other strategies should be considered because, in this study, there was no improvement observed with the interventions that were used.

The diabetes patients' education regarding self-care and the practice of specific exercises for the feet is an important practice and should be adopted by all health teams to prevent foot complications.

The study limitations include the absence of instruments to quantify the strength of the intrinsic muscles of the feet because they are small joints and they were evaluated manually [15]. Likewise, the peripheral pulse analysis was performed manually [29], which may have limited the results of that analysis. Nonetheless, all procedures were performed by the same examiner.

In conclusion, self-care associated with the guidelines for home exercises for the lower limbs in patients with type 2 DM are effective in maintaining and improving the alignment of the feet, mediolateral stability and prevention of complications.

## Supporting Information

Checklist S1 **CONSORT checklist.**
(PDF)

Protocol S1 **Trial protocol.**
(PDF)
