# Supplementary material for: Self-Care Associated with Home Exercises in Patients with Type 2 Diabetes Mellitus
Source: PLoS One. 2014 Dec 5;9(12):e114151. doi: 10.1371/journal.pone.0114151 (PMC4257588; doi:10.1371/journal.pone.0114151)
Supplement: Checklist S1 — CONSORT checklist. (PDF) [file pone.0114151.s001.pdf]

CONSORT 2010 checklist of information to include when reporting a randomised trial

Section/Topic	Item No	Checklist item	Reported on page No
**Title and abstract**			
	1a	Identification as a randomised trial in the title	No
	1b	Structured summary of trial design, methods, results, and conclusions (for specific guidance see CONSORT for abstracts)	Abstract
**Introduction**			
Background and objectives	2a	Scientific background and explanation of rationale	Introduction
	2b	Specific objectives or hypotheses	Introduction
**Methods**			Methods (Sample)
Trial design	3a	Description of trial design (such as parallel, factorial) including allocation ratio	Methods (Sample)
	3b	Important changes to methods after trial commencement (such as eligibility criteria), with reasons	No
Participants	4a	Eligibility criteria for participants	Methods (Sample)
	4b	Settings and locations where the data were collected	Methods (Sample)
Interventions	5	The interventions for each group with sufficient details to allow replication, including how and when they were actually administered	Methods (Sample) and Table 1
Outcomes	6a	Completely defined pre-specified primary and secondary outcome measures, including how and when they were assessed	Methods (Measures)
	6b	Any changes to trial outcomes after the trial commenced, with reasons	Methods (Measures)
Sample size	7a	How sample size was determined	Methods (Statistical Analysis)
	7b	When applicable, explanation of any interim analyses and stopping guidelines	No
Randomisation:			
Sequence generation	8a	Method used to generate the random allocation sequence	No
	8b	Type of randomisation; details of any restriction (such as blocking and block size)	No applicable
Allocation concealment mechanism	9	Mechanism used to implement the random allocation sequence (such as sequentially numbered containers) describing any steps taken to conceal the sequence until interventions were assigned	No applicable
Implementation	10	Who generated the random allocation sequence, who enrolled participants, and who assigned participants to interventions	No applicable

Blinding	11a	If done, who was blinded after assignment to interventions (for example, participants, care providers, those assessing outcomes) and how	No applicable
	11b	If relevant, description of the similarity of interventions	No
Statistical methods	12a	Statistical methods used to compare groups for primary and secondary outcomes	Methods (Statistical Analysis)Analysis
	12b	*Methods for additional analyses, such as subgroup analyses and adjusted analyses*	Methods (Statistical Analysis)
**Results**			
Participant flow (a diagram is strongly recommended)	13a	For each group, the numbers of participants who were randomly assigned, received intended treatment, and were analysed for the primary outcome	Methods (Figure 1)<br>Methods (Sample)
	13b	For each group, losses and exclusions after randomisation, together with reasons	Methods (Sample)
Recruitment	14a	Dates defining the periods of recruitment and follow-up	Methods (Sample)
	14b	Why the trial ended or was stopped	Methods (Sample)
Baseline data	15	A table showing baseline demographic and clinical characteristics for each group	Results (Table 2)
Numbers analysed	16	For each group, number of participants (denominator) included in each analysis and whether the by original assigned groups	Results and Measures
Outcomes and estimation	17a	For each primary and secondary outcome, results for each group, and the estimated effect size and its precision (such as 95% confidence interval)	Methods (Statistical Analysis)
	17b	For binary outcomes, presentation of both absolute and relative effect sizes is recommended	Table 3,4,5 – Results
Ancillary analyses	18	Results of any other analyses performed, including subgroup analyses and adjusted analyses, pre-specified from exploratory	Table 3,4,5 – Results
Harms	19	All important harms or unintended effects in each group (for specific guidance see CONSORT for harms)	Methods (Measures)
**Discussion**			
Limitations	20	Trial limitations, adressing sources of potencial bias, imprecision, and, if relevant, multicity of	Discussion
Generalisability	21	Generalisability (external validity, applicability) of the trial findings	Discussion
Interpretation	22	Interpretation consistent with results, balancing benefits and harms, and considering other	Discussion
**Other information**			
Registration	23	Registration number and name of trial registry	Abstract
Protocol	24	Where the full trial protocol can be accessed, if available	Abstract
Funding	25	Sources of funding and other support (such as supply of drugs), role of funders	Funding
