# Supplementary material for: Self-Care Associated with Home Exercises in Patients with Type 2 Diabetes Mellitus
Source: PLoS One. 2014 Dec 5;9(12):e114151. doi: 10.1371/journal.pone.0114151 (PMC4257588; doi:10.1371/journal.pone.0114151)
Supplement: Protocol S1 — Trial protocol. (PDF) [file pone.0114151.s002.pdf]

TRIAL STUDY PROTOCOL RESEARCH ETHICS COMMITTEE OF THE FEDERAL UNIVERSITY OF ALFENAS.

All individuals initially were avaliated by the same examiner before and after the follow-up period and the practical guidelines on the management and prevention of diabetic foot ulcerations were followed:

1. Initially we provided information about the study's objectives, which the volunteers agreed on signing the term before participating.
2. Sociodemographic and health factors were collected, including age, gender, BMI, duration of diabetes, glycemic control type, and factors associated with diabetes, such as smoking, alcohol consumption, hypertension,
3. Avaliation on the skin aspects, toe deformities,
4. Avaliation on the muscle strength of the toes' flexors and extensors, hallux extensor and eversion,
5. Avaliation on the sensitivity (Semmes-Weinstein 10 g),
6. Avaliation on the joint mobility
7. Avaliation on the circulation aspects (the presence of edema as well as tibial and dorsal pedis pulses).
8. Avaliation on the neuropathy symptoms score (NSS)
9. Collected photographs of the feet and ankles' anterior, posterior and plant for photogrammetric analysis.
10. Static as well as dynamic baropodometric records were taken with the aid of a FootWork Pressure Plate (FPP) (IST Informatique, France).
11. All individuals initially received individual verbal guidance as well as an explanatory leaflet about self-care and lower limb exercises:

11.1.Self-care:

- Use of closed shoes,
- Do not walk barefoot,
- Cut the nails properly
- Use mirror for self-examination,
- Proper drying of the feet,
- Wearing cotton socks,
- Use moisturizer on legs,
- Non-application of moisturizer between toes,
- Do not use abrasives or sandpaper

11.2 Exercise:

- Dorsiflexion and plantar flexion, lying (pumping exercise). Verbal Command: "Lying, raise and lower your foot tip 30 times".
- Feet circumduction, lying (pumping exercise). Verbal Command: "Make legs rotate 30 times to the right, then 30 times to the left".
- Dorsiflexion and plantar flexion, sitting (pumping exercise). Verbal Command: "While seated, lift your heels off the ground and lower and lift the tips of the feet 30 times".
- Stretching flexors of the toes. Verbal Command: "Rest your feet flat and supported, stretch your toes to the maximum and count to 50".
- An exercise to strengthen the intrinsic muscles of the feet using a towel - Verbal Command: "Extend a large towel on the ground, rest both feet on it, and then pull the toes with the towel".
- Proprioceptive exercise of the foot plant with a curly ball. Verbal Command: "For two minutes for each foot, slide your feet on a curly short small ball".

- Exercise to strengthen the intrinsic muscles of the feet with the use of marble. Verbal Command: "Hold 30 marbles with the toes of each foot".
- Free active exercise for hip abductors in standing position. Verbal Command: "While standing, close your legs for 30 repetitions".
- Free active exercise for hip abductors in standing position. Verbal Command: "While standing, open the legs for 30 repetitions”.
- Stretching triceps surae. Verbal Command: "Standing with one foot forward and one back and the heels in contact with the ground, count to 50".

12. follow-up examinations for 10 consecutive months which they were instructed to return at least once per month.
13. Reavaliation
14. Analyze the images to verify the angular measurements (CorporisPro® - Datahominis Technology, Brazil), and Postural Assessment Software (SAPo) was used to analyze the linear measurements of the plantar arch.
15. Data Analysis and Statistical Analysis
